# Application of non-invasive ICP waveform analysis in acute brain injury: Intracranial Compliance Scale

**DOI:** 10.1186/s40635-023-00492-9

**Published:** 2023-01-27

**Authors:** Gustavo Frigieri, Chiara Robba, Fábio Santana Machado, Joao A. Gomes, Sérgio Brasil

**Affiliations:** 1 Medical Investigation Laboratory 62, School of Medicine, University of São Paulo, São Paulo, Brazil; 2 Division of Neurosurgery, Department of Neurology, School of Medicine, University of São Paulo, São Paulo, Brazil; 3 Department of Intensive Care, Universitá Degli Studi Di Genoa, Genova, Italy; 4 Department of Intensive Care, Hospital Sírio Libanês, São Paulo, Brazil; 5 Cerebrovascular Center, Neurologic Institute, Cleveland Clinic, Cleveland, OH USA

## Dear editor,

Various non-invasive monitoring techniques have been developed in recent years seeking to minimize the burden that repeated imaging and invasive procedures may inflict on the journey of patients with acute brain injuries (ABI). Furthermore, the combination of these methods may theoretically enhance their predictive value for functional outcomes, as well as the detection of alterations in intracranial compliance (ICC) and intracranial hypertension [1].

One such emerging technique is the monitoring of ICC based on intracranial pressure waveform (ICPW) parameters [2–4]. ICC impairment has been linked to both elevations in the P2/P1 ratio (namely, a tidal peak (P2) amplitude taller than the upstroke peak (P1) amplitude on the ICPW), and to prolongations of the time interval from pulse triggering until maximum amplitude on the ICPW is reached (time-to-peak [TTP], Additional file 1: Figure S1) [4].

In the current investigation, we report our preliminary experience with a black box algorithm that derives a composite index of ICC (Intracranial Compliance Scale—ICS) from non-invasive ICP waveform surrogates. Our primary aim was to evaluate the ICS ability to detect elevations in ICP. Secondarily, we sought out to correlate ICS with early in-hospital mortality (i.e., 7 days) since this time-period correlated best with deaths related to intracranial hypertension in our cohort.

In the present report, we revisited data from 72 patients with ABI. The inclusion criteria were ABI in need of invasive ICP monitoring. Included patients were assessed within the first 5 days following hospital admission with a skull deformation extensometer (Brain4care Corp, São Carlos, Brazil), able to register the non-invasive ICPW [5]. Therefore, ICP values were correlated with non-invasive ICPW parameters (P2/P1 ratio and TTP) [4, 5]. This prospective monocentric case series complied with the STROBE guidelines for observational studies and received local ethics committee approval. More detailed methodological, clinical, and demographic data are presented elsewhere [5]. Unfavorable outcome was defined as early in-hospital mortality, whereas patients still alive at the 7-day mark were considered favorable. As previously published, admission Glasgow Coma Scale, and disease severity scores (Simplified Acute Physiology Score 3) did not differ between groups. However, intracranial pressure was significantly higher among patients with early in-hospital mortality [5].

**Fig. 1:**
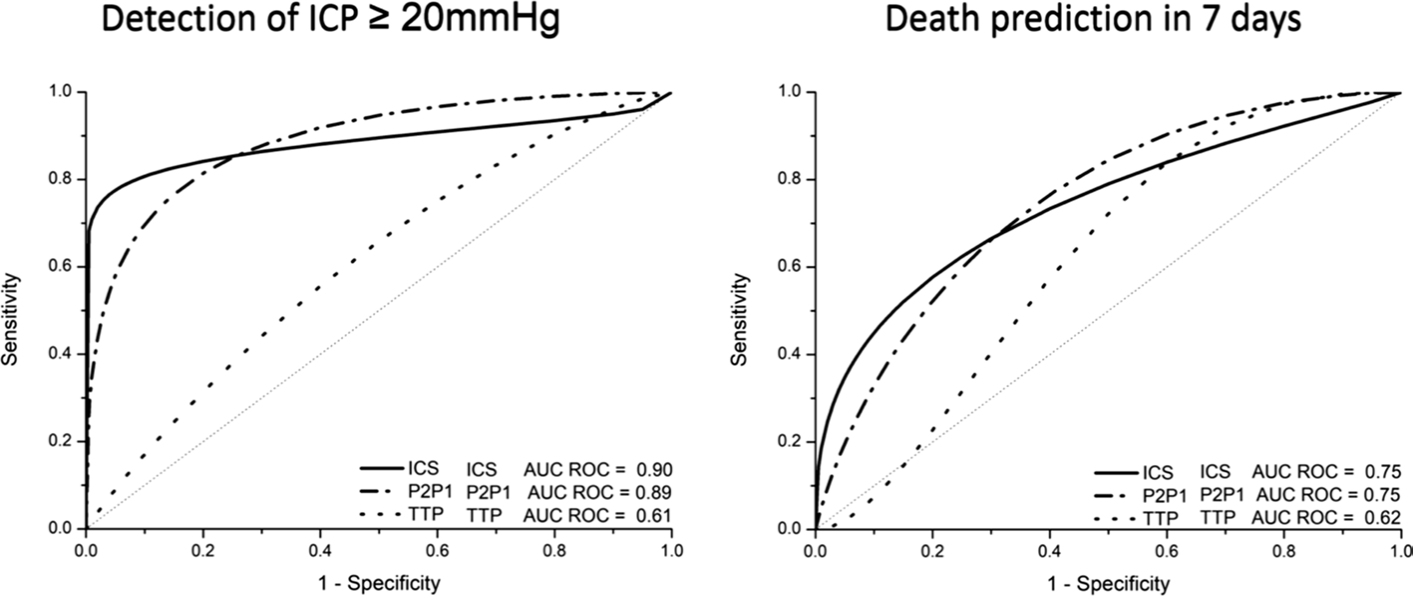
Area under receiver operator curve (AUROC) analysis of P2/P1 ratio, TTP and ICS to intracranial hypertension detection (left) and death prediction (right). ICP: intracranial pressure, ICS: Intracranial Compliance Scale, TTP: time-to peak. AUROC analysis—J Hopkins Web Tool (Johns Hopkins University. Available from: http://www.jrocfit.org.)

The ICS algorithm was based on a progressive 4-point score (Anaconda package coded in Pycharm, available at https://www.jetbrains.com). ICS score 0 was associated with a negative predictive value of 100%, whereas ICS score 3 held a positive predictive value of 100% (Additional file 2: Table S1) for ICC impairment. Compared to ICPW individual parameters P2/P1 ratio and TTP, ICS increased the area under the receiving operator curve and reduced the confidence interval to detect patients with intracranial hypertension (Fig. 1). Notably, no early death patients had an ICS score of 0, whereas none of the survivors had an ICS score of 3 (Additional file 3: Figure S2).

Our preliminary results suggest that the ICS is a reliable tool for the detection of ICC alterations. Furthermore, it had better overall performance than individual analysis of ICPW peak amplitudes or time intervals for the prediction of the pre-specified outcome measures. Since the ICS can be calculated continuously and automatically in real time at the bedside, translation to medical management of patients with ABI can be achieved seamlessly. Given limitations related to sample size, a multi-center clinical cohort validation study seems warranted.

## Supplementary Information

**Additional file 1. Supplemental figure 1**. Intracranial pressure waveform (ICPW) parameters based on peak amplitudes and time interval. Morphology examples of standard and impaired ICPW. Amp: amplitude, P1: upstroke peak, P2: tidal peak, TTP: time to peak.

**Additional file 2. Supplemental table 1**. Sensitivity, specificity, area under the receiving operator curve (accuracy), positive predictive value (PPV) and negative predictive value (NPV) analysis for TTP, P2/P1 and ICS for the detection of intracranial hypertension (ICP ≥ 20mmHg). ICS: intracranial compliance scale, TTP: time-to-peak. Statistical analysis using Scikit package, Python 3.7.

**Additional file 3. Supplemental figure 3**. TTP, P2/P1 ratio and ICS scatterplots to intracranial hypertension (ICP ≥ 20 mmHg, below) and short-term outcomes (above). ICS: intracranial compliance scale, FO: favorable outcome (survivors), UO: unfavorable outcome (death), TTP: time-to-peak. Boxplot and Anova analysis calculated with algorithms Python 3.7.
