# Supplementary material for: Application of non-invasive ICP waveform analysis in acute brain injury: Intracranial Compliance Scale
Source: Intensive Care Med Exp. 2023 Jan 27;11:5. doi: 10.1186/s40635-023-00492-9 (PMC9880126; doi:10.1186/s40635-023-00492-9)
Supplement: Supplementary file 1 — Additional file 1. Supplemental figure 1. Intracranial pressure waveform (ICPW) parameters based on peak amplitudes and time interval. Morphology examples of standard and impaired ICPW. Amp: amplitude, P1: upstroke peak, P2: tidal peak, TTP: time to peak. [file 40635_2023_492_MOESM1_ESM.docx]

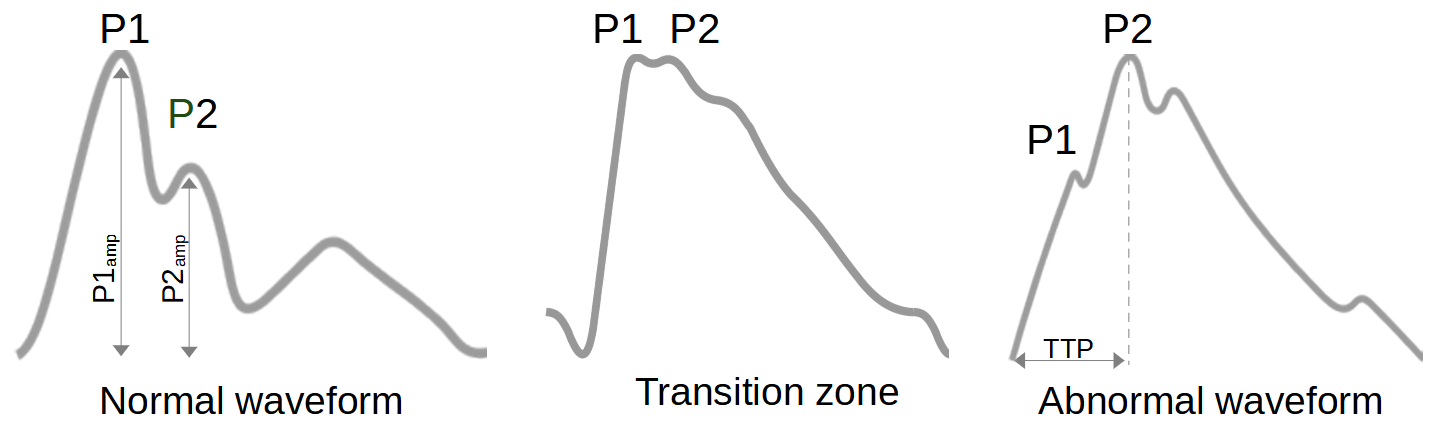


Supplemental figure 1. Intracranial pressure waveform (ICPW) parameters based on peak amplitudes and time interval. Morphology examples of standard and impaired ICPW. Amp: amplitude, P1: upstroke peak, P2: tidal peak, TTP: time to peak.
