# Supplementary material for: Application of non-invasive ICP waveform analysis in acute brain injury: Intracranial Compliance Scale
Source: Intensive Care Med Exp. 2023 Jan 27;11:5. doi: 10.1186/s40635-023-00492-9 (PMC9880126; doi:10.1186/s40635-023-00492-9)
Supplement: Supplementary file 2 — Additional file 2. Supplemental table 1. Sensitivity, specificity, area under the receiving operator curve (accuracy), positive predictive value (PPV) and negative predictive value (NPV) analysis for TTP, P2/P1 and ICS for the detection of intracranial hypertension (ICP ≥ 20mmHg). ICS: intracranial compliance scale, TTP: time-to-peak. Statistical analysis using Scikit package, Python 3.7. [file 40635_2023_492_MOESM2_ESM.docx]

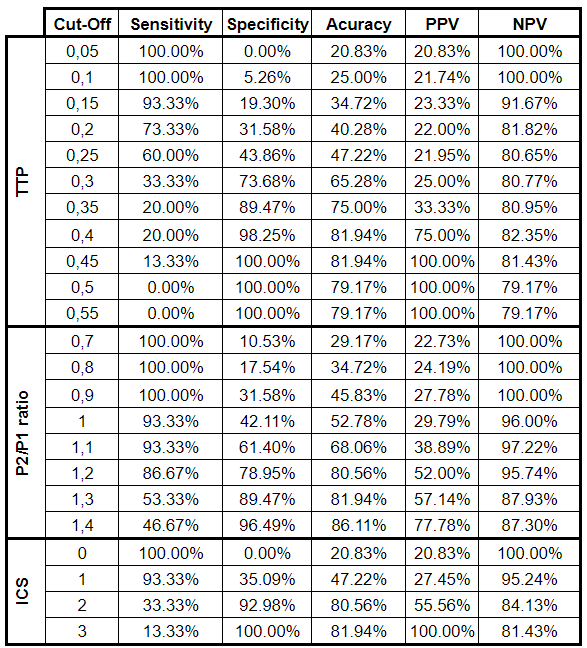


Supplemental table 1. Sensitivity, specificity, area under the receiving operator curve (accuracy), positive predictive value (PPV) and negative predictive value (NPV) analysis for TTP, P2/P1 and ICS for the detection of intracranial hypertension (ICP ≥ 20mmHg). ICS: intracranial compliance scale, TTP: time-to-peak. Statistical analysis using Scikit package, Python 3.7.
