# Supplementary material for: Application of non-invasive ICP waveform analysis in acute brain injury: Intracranial Compliance Scale
Source: Intensive Care Med Exp. 2023 Jan 27;11:5. doi: 10.1186/s40635-023-00492-9 (PMC9880126; doi:10.1186/s40635-023-00492-9)
Supplement: Supplementary file 3 — Additional file 3. Supplemental figure 3. TTP, P2/P1 ratio and ICS scatterplots to intracranial hypertension (ICP ≥ 20 mmHg, below) and short-term outcomes (above). ICS: intracranial compliance scale, FO: favorable outcome (survivors), UO: unfavorable outcome (death), TTP: time-to-peak. Boxplot and Anova analysis calculated with algorithms Python 3.7. [file 40635_2023_492_MOESM3_ESM.docx]

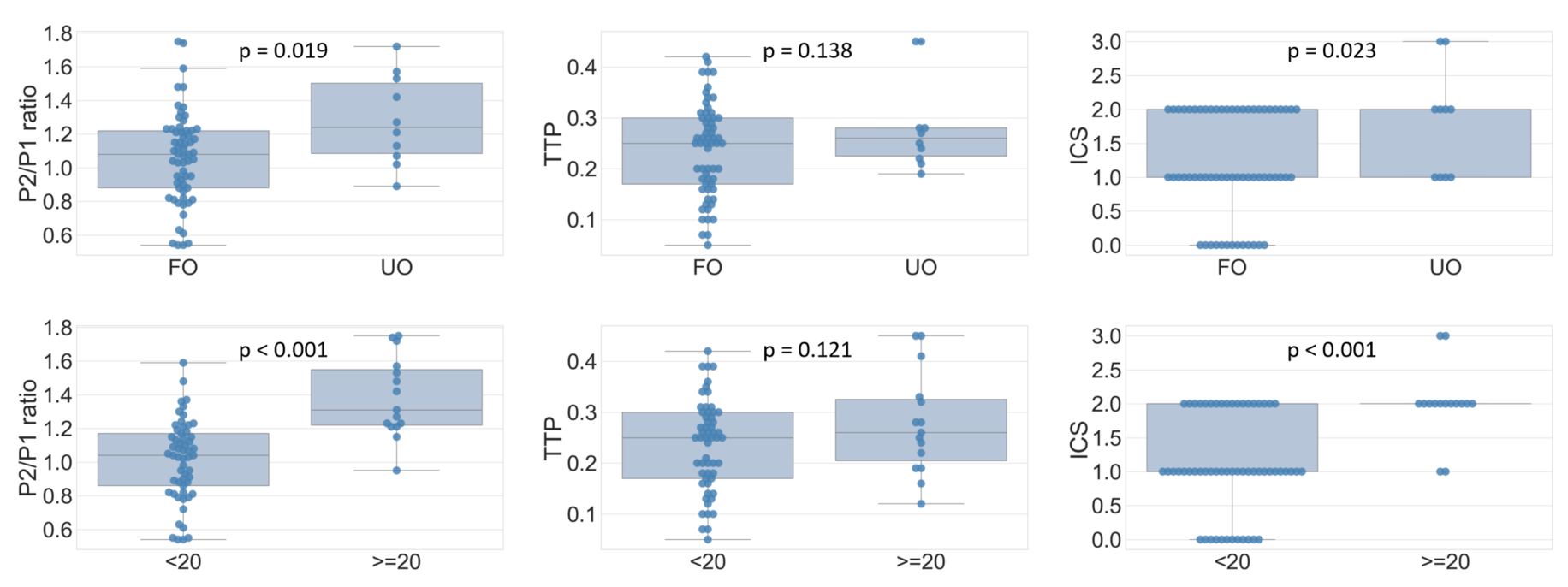


Supplemental figure 3. TTP, P2/P1 ratio and ICS scatterplots to intracranial hypertension (ICP ≥ 20 mmHg, below) and short-term outcomes (above). ICS: intracranial compliance scale, FO: favorable outcome (survivors), UO: unfavorable outcome (death), TTP: time-to-peak. Boxplot and Anova analysis calculated with algorithms Python 3.7.
